# A Review of Total Hip Arthroplasty Comparison in FNF and OA Patients

**DOI:** 10.1155/2021/5563500

**Published:** 2021-09-17

**Authors:** Jakub Szczesiul, Marek Bielecki

**Affiliations:** ^1^Department of Orthopedic, Traumatology and Hand Surgery, Medical University of Białystok, Białystok, Poland; ^2^Department of Orthopedic, Traumatology and Hand Surgery, Uniwersytecki Szpital Kliniczny w Białymstoku, Białystok, Poland

## Abstract

**Background:**

Worldwide, total hip arthroplasty (THA) has become one of the most commonly performed surgical procedures. Femoral neck fracture (FNF) and osteoarthritis (OA) are two of the medical conditions necessitating a hip replacement, most frequently carried out. The preoperative and postoperative pathways for patients suffering from these two diseases differ, yet worldwide, many national healthcare systems underestimate or misinterpret the (more than nuanced) care plan differences of the two. *Factors and Criteria*. Analyzed material was gathered from studies published between 2013 and 2019. Various strands of data demographics, comorbidities, and complications, as well as treatment outcomes, were tabulated to compare and contrast THA patients suffering from FNF and OA to collate their findings. Outcomes were cross-checked and validated for reliability and then were presented in a table format.

**Results:**

All five retrospective cohort studies fitted the required criteria for inclusion in this work, four US-based study groups and one European-based study group. Data were gathered from three separate databases. The “average” FNF patient is 76.8 years old. There was a 68.96% female probability. The “average” OA patient is 69.15 years old. There was a 5.24% female probability. 59.57% operated for athrosis, and only 34.63% operated for fracture which received grade lower than the third in the American Society of Anaesthesiologist (ASA) classification. There was more than 3 times higher prevalence of complications in the trauma group. FNF patients' hospitalization was approximately 3 days longer. On average, 3.7% of patients operated for trauma and 1.5% of patients with elective THA required a second surgery. 6.57% FNF and 2.93% OA patients had unplanned readmission.

**Conclusions:**

In general, patients who suffer a femoral neck fracture are an extremely fragile group. They require additional perioperative and postoperative care. To meet these desired expectations, more FNF cost-comprehensive systems need to be initiated.

## 1. Introduction

### 1.1. Rationale

Hip arthroplasty which may be divided into total hip arthroplasty (THA) and hemiarthroplasty (HA) is a procedure that allows to replace damaged parts of the hip joint, such as the femoral head and neck or hip acetabulum, with artificial ones. The materials that compose the components are mostly titanium, titanium-cobalt alloy, stainless steel, or ceramic and are characterized by good biocompatibility.

Total hip arthroplasty (THA), due to its application in both osteoarthritis (OA) and displaced femoral neck fracture (FNF), has become a frequent orthopedic procedure worldwide. Complex medical care that prolongs patient's lifespan which leads to increased incidence of fragility hip fracture [1–3] makes the procedure even more commonly utilized.

Intra-articular hip fracture rates among top three of all hip fractures [4]. FNF is characterized by poor healing which necessitates hip arthroplasty. New research shows the advantage of THA over hemiarthroplasty in FNF treatment in terms of clinical results and reoperation rate, despite a higher incidence of dislocation in THA [4–6]. Fortunately, the latest studies indicate that dual-mobility total hip arthroplasty (DM THA) nullifies the dislocation disadvantage, and thus, THA remains a preferred treatment method in FNF in active elderly patients [7, 8]. The same procedure is also performed to increase hip mobility and relieve pain in hip joint osteoarthrosis. Even though endoprosthesis components and the conduct of the operation do not differ in THA for FNF and OA, patients' demographics, comorbidities, complications, or treatment outcomes vary in both groups [9–14].

This paper aims to gather and summarize publications related to THA comparison between patients suffering from femoral neck fracture and hip osteoarthritis in order to improve the understanding of the differences between these two groups.

### 1.2. Objectives

This is a traditional narrative review of articles published since 2012 comparing FNF and OA patients undergoing THA. The objective was to compare (1) preoperational status, (2) perioperation procedures performed before and after the surgery, and (3) complication rate in those groups. Furthermore, this study intends to highlight (4) strengths and weaknesses of gathered evidence and (5) guidelines for future research.

## 2. Materials and Methods

### 2.1. Data Source

**Figure 1:**
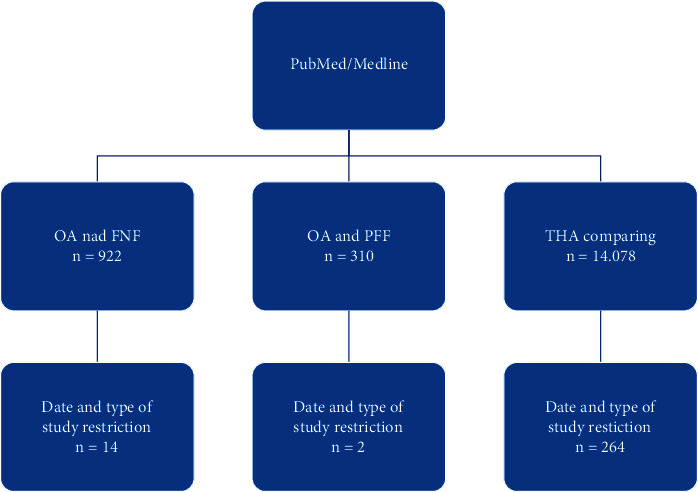
Results of scientific research findings based on the used phrase. OA: osteoarthrosis; FNF: femoral neck fracture; PFF: proximal femur fracture; THA: total hip arthroplasty.

The PubMed/MEDLINE database was used to gather observational studies regarding the comparison of THA in FNF and OA. Article selection was conducted using words “*total hip arthroplasty comparing*,” “*osteoarthritis and femoral neck fracture*,*”* and “*osteoarthritis and proximal femoral fracture*.” In order to meet inclusion criteria, the paper published date should not be older than 2012, study design should be observation study with retrospective cohort; also, a comparison of both groups' epidemiology and postoperative outcome was mandatory. The last search was carried out in June 2021 and resulted in finding 5 studies, including publications written by Schairer et al. [9], Charette et al. [10], Adam et al. [11], Qin et al. [12], and Le Manach et al. [13]. The selection process was illustrated on the graph (Figure 1).

The articles were written based on the data from 3 different databases. Three of them utilize the National Surgical Quality Improvement Program (NSQIP) database, one the National Hospital Discharge Database (NHDS), and one the French Hospital Discharge Database (FHDD). Four of the selected studies were based on the American and one on the European population (Table 1). Data concerning the patients' demographics, comorbidities, complications, and treatment outcomes were extracted.

### 2.2. Study Population

In all studies, patient populations were gathered based on the International Statistical Classification of Diseases and Health Related Problems (ICD 9) procedures. Authors utilized ICD 9 715.15, with the assist of CPT 2710 to determine patients' groups. Reoperation, polytrauma, and hip cancer cases were excluded from research. Even though each publication is a retrospective cohort study, the authors present different approaches to data selection, statistical analysis, or group observation period. For example, only Sasson et al. and Le Manach et al. decided to establish an age cutoff limit at 45 years. There were no data regarding minimal age research inclusion in the remaining works. THA as treatment for either FNF or OA was examined as the binary variable. In addition, in all listed studies, demographic data such as age, body mass, and comorbidities were included. Moreover, information concerning length of stay, home dismissal, and unplanned readmission was provided. Last but not least, some articles presented other additional data such as blood loss, postoperation function status, operation duration, preoperation transfusions, and type of anesthesia.

### 2.3. Statistical Analysis

Information concerning epidemiology, comorbidities, perioperation procedures, and complications was compared. Arithmetic average, standard deviation, and variance were calculated. A majority of gathered data were presented in tables. Additional analyses were performed on population matching cohorts. Results were described via diagrams.

## 3. Results

Factors that were studied in mentioned publications can be divided into four groups: demographic, comorbidities, complications, and outcome. The approaches in data collection chosen by the authors were not identical, yet similarities in their study design allow some degree of data comparison.

Patients' demographic: based on elaborated research, an average femoral fracture patient is 76.8 years old, and 68.96% of them are female. 55.24% OA patients were women, and OA patient average age was 69.15 years (Table 2). In Charette's work, there were no data concerning average age; instead, patients were divided into additional groups, with age above and below 70.

Patient medical records show a higher prevalence of comorbidities in FNF groups with the exception of obesity, which was more common in OA groups (Table 3). Most of the research papers used the ASA score to evaluate patient preoperative risk factors. The exception was Sasson et al. and Le Manach et al. Sasson et al. utilized the Deyo comorbidity score, and in research from France, no physical status classification has been used. The authors tabulated most common comorbidities in OA/FNF patients including their frequency instead. Based on publications written by Charles et al. and Charette et al., on average, 34.63% of patients undergoing surgery for femoral neck fracture and 59.57% of arthrosis patients were classified as ASA I + II. 65.37% treated for FNF and 40.03% operated for OA were classified as ASA III + IV. Research shows a higher prevalence of all comorbidities in the FNF group with the exception of obesity, although hypertension, according to some research studies [12, 14], may also be more common in the OA patient group.

Factors taken into consideration upon complication assessment in the discussed publications are not uniform; therefore, some data cannot be compared. Moreover, four out of five authors performed propensity matching on the queried groups. A. Sasson et al. calculated mortality in FNF to be 1.8% and in OA to be 0.2%, risk of pulmonary embolus in FNF to be 0.8% and in OA to be 0.3%, and risk of infection in FNF to be 1.7% and in OA to be 0.3%. Dislocation rate is also seven times higher in the fracture population, which is also more prone to unstable luxation [15]. Complications described above are restricted to hospitalization, and no propensity score matching was performed on these data. C.D. Qin et al. stated that 11.1% of FNF and 3% of patients with OA suffer in-hospital complications. After matching the cohorts, the numbers were 10.7% for FNF and 4% for OA patients. The study also indicates a significant difference in function status after the surgery (2% vs. 9.2%) in the matched population. Y. Le Manach et al. also compared postoperative outcomes in unmatched and matched study populations. In-hospital mortality was rated 3.42% and 0.18% in the unmatched population and 1.82% and 0.31 in the matched cohort in FNF and OA, respectively. The authors detailed myocardial infarction (0.36% vs. 0.22%), heart failure (5.22% vs. 0.77%), stroke (0.39% vs. 0.15%), renal failure (0.65% vs. 0.30%), and sepsis (0.27% vs. 0.09%), and the data consider the matched population study. Wiliam W. et al. in publication from 2016 described overall complication rate in the propensity score-matched cohort as 15% vs. 6% and surgical complication rate as 4% vs. 2% for FNF vs. OA populations. Chalette R. S. et al. also conducted research employing the propensity matching method. Their findings compared to the results of William W. et al. are demonstrated in Table 4.

In outcome analysis, all researchers took into consideration length of stay in-hospital and nonhomebound discharge and, except for Dr. Sasson, performed reoperations (Table 5). Moreover, in two publications, an additional endpoint, reoperation rate, was included. Based on the studies, the average length of stay for patients with FNF is almost 8 days, while OA patients stay in the ward only for 5 days on average. More than 67% of patients with hip fracture were discharged to another medical or caring facility, whilst 60% operated for hip osteoarthrosis went back home after the procedure. The described trends may also be a risk factor. According to Michael et al., a discharge to inpatient facilities after total hip arthroplasty is associated with increased postdischarge morbidity [16]. Often, requirement for additional postoperative care was described in Qin et al.'s study. Before the procedure, 9% of FNF and only 2% of OA patients were classified as functional dependent [13]. Readmissions are also more common for the trauma group. More than 6.5% of fractures had additional hospitalizations, and they were twice as much as for primary THA. Furthermore, based on the publication from 2016 to 2019, on average, 3.5% of FNF patients and 1.5% of OA patients required reoperation. It may be related to the increasing probability of periprosthetic fracture in the trauma group as both populations' level of BMD differs not only in the postoperation period but also in the preoperation period [17–19].

### 3.1. Significance

Based on elaborated research,

- average FNF patient is 7.64 years older than the OA patient.
- There is statistically significant predominance to a female gender in the trauma group.
- Fracture patients display a higher prevalence of comorbidities with the exception of obesity to which the primary THA group is more prone to.
- FNF patients usually require longer hospitalizations and more frequent blood transfusions.
- Trauma group is more than 2 times more prone to complications including respiratory complications or even death than the OA group. Furthermore, the studies indicate that “fractures” required more frequent reoperations.
- FNF patients display worse postoperation functional status and are more often discharged to another medical or caring facility.

## 4. Discussion

Five publications were included in this study, and despite varying approaches to the subject, they all agree on the fact that FNF and OA patient groups differ in many ways. One could say that the type of treatment might be the only common feature. It is noticeable in demographics, comorbidities, and laboratory results, as well as in postoperative outcomes or complications [14, 20]. Authors of all mentioned publications agree that patients with hip fracture require additional care and have poorer prognosis than primary THA patients. Schairer et al. [10] indicated that patient death after surgically treated hip fracture is almost 10 times more probable than after hip OA operation. That study also compared complication frequency in both groups and showed almost 3 times higher prevalence of trauma in patients. Other authors described similar observations. Poor treatment outcome can be related to FNF patient group senile age and numerous comorbidities, but R.S. Charette et al. in their study [12] conducted propensity matching and multivariate analysis and came to a conclusion that hip fracture is an independent risk factor. Since the groups and their main diseases differ, despite similar surgical treatment, distinct in-hospital and often posthospital care is required. According to William W. et al., the trauma group needs, on average, 10 times more blood transfusions and 2 more days of preoperative preparations than the primary THA group [11]. Taking length of hospital stay into consideration, the percentage of nonhomebound discharged patients, and other mentioned factors, FNF patients appear more cost-intensive. Unfortunately, most medical facilities are paid for procedures; therefore, both groups are paid for in a similar fashion. This reasoning may indicate unjust THA financing policies whether it is a bundle payment or restricted by a hospital-government contract on the defined number of hip arthroplasty procedures. The problem was presented in previous subject-related publications [9–13], and all authors agree that a different system should be established. Additional studies should be held to determine the cost of prolonged THA hospitalization with particular emphasis on FNF patients.

The study has several limitations. Firstly, data in analyzed studies were not uniform. Secondly, study construction differed between publications. Observation period, population matching, and researched complications were not homogenous; therefore, calculations based on the data may only exhibit trends. Finally, no full statistical analysis was performed on the gathered material.

In summary, THA provides optimal treatment for both OA and FNF in active elderly patients. Because mentioned diseases vary in many aspects, their treatment should benefit from a different perioperative approach. In order to maintain best medical care, a more cost-comprehensive system should be established, a system that would penalize facilities performing THA for FNF instead for OA.

## Figures and Tables

**Table 1:** Study design comparison.

Author, publication year	Study period	Patient group	Study design	Database	Population matching	Observation period
Sasson A., 2012/2013	1990–2007	174.641 FNF 2.160.061 OA	Retrospective cohort	NHDS	No	Hospitalization

Wiliam W., 2016	2007–2013	953 FNF 41.739 OA	Retrospective cohort	NSQIP	Yes	30 days after discharge

Le Manach Y., 2015	2010–2013	319.804 FNF 371.191 OA	Retrospective cohort	FHDD	Yes	Hospitalization + readmission 72 h

Charles D., 2016	2011–2014	1.580 FNF 58.302 OA	Retrospective cohort	NSQIP	Yes	30 days after discharge

Charette R. S., 2019	2008–2016	4.266 FNF 135.013 OA	Retrospective cohort	NSQIP	Yes	30 days after discharge

**Table 2:** Demographic comparison.

Author, publication year	Patient group	Average age	Female gender
Sasson A., 2012/2013	174.641 FNF 2.160.061 OA	79.1 FNF 68.4 OA	75.3% FNF 55.0% OA

Wiliam W., 2016	953 FNF 41.739 OA	73.4 FNF 65.0 OA	58.3% FNF 55% OA

Le Manach Y., 2015	319.804 FNF 371.191 OA	81.7 FNF 70.2 OA	74.8% FNF 55.6% OA

Charles D., 2016	1.580 FNF 58.302 OA	73.0 FNF 66.0 OA	67.9% FNF 55.8% OA

Charette R. S., 2019	4.266 FNF 135.013 OA	—	68.5 FNF 54.8 OA

**Table 3:** Comorbidities' comparison.

Publication	Classification	FNF	OA	Obesity^*∗*^
Sasson A., 2012/2013	DCS none	55.1%	75.9%	No data
	DCS M + S	44.9%	24.1%	No data

Le Manach Y., 2015	No ASA classification			FNF 2.1%
				OA 10.3%

Wiliam W., 2016	ASA I + II	35,00%	60,00%	FNF 21%
	ASA III + IV	65,00%	40,00%	OA 45%

Charles D., 2016	ASA I + II	35.3%	60.5%	FNF 25.7^∗∗^
	ASA III + IV	64.7%	39.5%	OA 30.3^∗∗^

Charette R. S., 2019	ASA I + II	33.6%	58.2%	FNF 23.8%
	ASA III + IV	66.4%	41.8%	OA 46.3%

^*∗*^Patient body mass index above 30 kg/m^2^. ^∗∗^Average BMI in the FNF/OA population.

**Table 4:** Selected complications' comparison.

Complication	Group	Study	
		William W. (%)	Chalette R. S. (%)
Mortality	FNF	3	1.8
	OA	0	0.3

Wound infection	FNF	1.5	1
	OA	1.4	1

Respiratory complications	FNF	3	1
	OA	0	0.3

**Table 5:** Outcome comparison.

Outcome	Publication	Sasson A., 2012/2013	Wiliam W., 2016	Le Manach Y., 2015	Charles D. Qin, 2016	Charette R. S., 2019
	Disease					
Readmission	FNF	No data	4%^*∗*^	0.44%^∗∗∗^	7.7%	8%
	OA	No data	2%^*∗*^	1.22^∗∗^	3.3%	3.5%

Nonhomebound discharge	FNF	79.3%	67%^*∗*^	65.2%	63.4%	61%
	OA	49.2%	55%^*∗*^	40%	25.9%	22%

Average length of stay (days)	FNF	9.1 days	6 days	12.1 days	4.5 days	53.8%^∗∗^
	OA	5.1 days	4 days	7.8 days	3 days	7.5%^∗∗^

Reoperation	FNF	No data	3%^*∗*^	No data	No data	4.4%
	OA	No data	1%^*∗*^	No data	No data	2%

^*∗*^Matched cohort value. ^∗∗^More than 5 days. ^∗∗∗^Within 72 hours.

## Data Availability

The research was based on the data extracted from the studies mentioned in Table 1. No other data were used to support this study.
